# Topical Delivery of Hedgehog Inhibitors: Current Status and Perspectives

**DOI:** 10.3390/ijms232214191

**Published:** 2022-11-16

**Authors:** Kristian Kåber Pedersen, Maria Helena Høyer-Hansen, Thomas Litman, Merete Hædersdal, Uffe Høgh Olesen

**Affiliations:** 1 Department of Dermatology, Copenhagen University Hospital—Bispebjerg and Frederiksberg, 2400 Copenhagen, Denmark; 2 Molecular Biomedicine, LEO Pharma A/S, 2750 Ballerup, Denmark; 3 Department of Immunology and Microbiology, University of Copenhagen, 2200 Copenhagen, Denmark

**Keywords:** keratinocyte carcinoma, basal cell carcinoma, hedgehog inhibitors, smoothened inhibitors, vismodegib, sonidegib, topical delivery

## Abstract

Systemic treatment with hedgehog inhibitors (HHis) is available to treat basal cell carcinomas but their utility is limited by adverse effects. Topical delivery methods may reduce adverse effects, but successful topical treatment depends on sufficient skin uptake, biological response, and time in tumor tissue. The aim of this review was to evaluate the current status of topical HHi delivery for BCCs and discuss barriers for translating systemic HHis into topical treatments. A literature search identified 16 preclinical studies and 7 clinical trials on the topical delivery of 12 HHis that have been clinically tested on BCCs. Preclinical studies on drug uptake demonstrated that novel formulations, and delivery- and pre-treatment techniques enhanced topical HHi delivery. Murine studies showed that the topical delivery of sonidegib, itraconazole, vitamin D₃ and CUR-61414 led to biological responses and tumor remission. In clinical trials, only topical patidegib and sonidegib led to at least a partial response in 26/86 BCCs and 30/34 patients, respectively. However, histological clearance was not observed in the samples analyzed. In conclusion, the incomplete clinical response could be due to poor HHi uptake, biodistribution or biological response over time. Novel topical delivery techniques may improve HHi delivery, but additional research on cutaneous pharmacokinetics and biological response is needed.

## 1. Introduction

**Figure 1:**
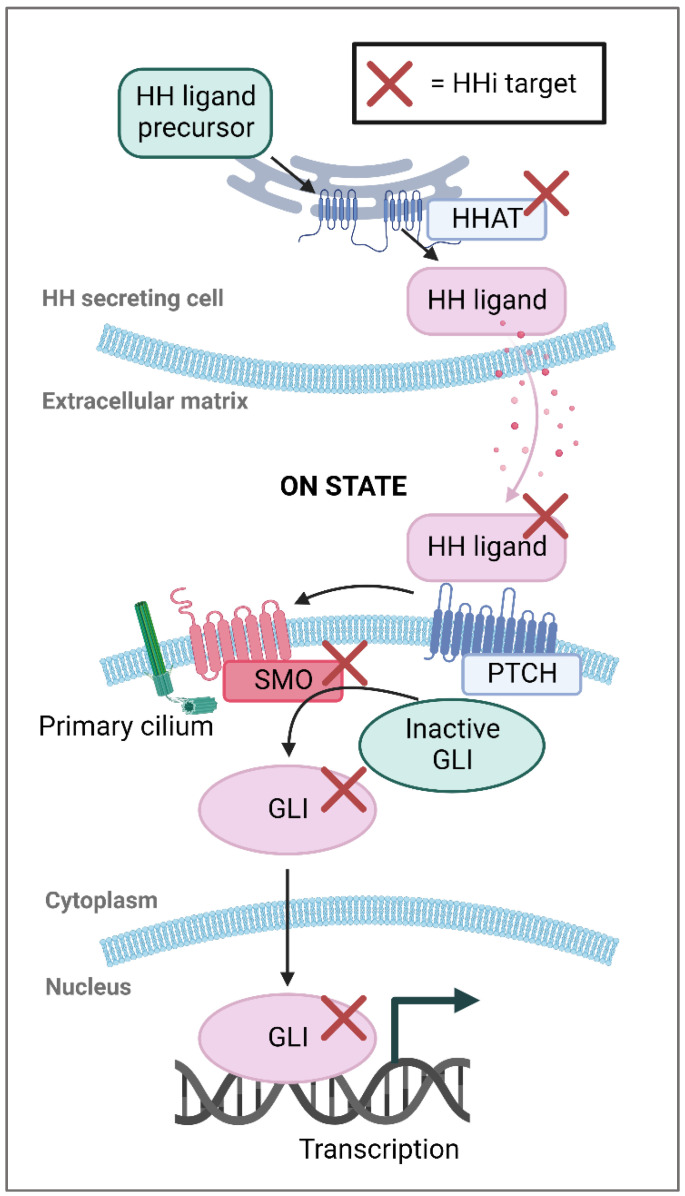
The main components of the hedgehog pathway. Membrane proteins are either blue or red, inactive proteins are green and active proteins are colored pink. The red crosses indicate target proteins for hedgehog inhibitors (HHis). In canonical hedgehog (HH) signaling, HH ligands are modified by hedgehog acyltransferase (HHAT) and released from HH-secreting cells. HH ligands then bind to the cell membrane protein patched (PTCH), which leads to release of smoothened (SMO). SMO moves to the primary cilium where it prevents breakdown of GLI family zinc finger proteins (GLI) allowing them to translocate to the nucleus and promote expression of HH-signaling target genes. In basal cell carcinomas (BCCs), inactivating *PTCH1* mutations are most common (70–90%) followed by activating *SMO* mutations (10–20%). Created with BioRender.com.

Keratinocyte carcinomas are the most common human malignancies and include cancers that develop in both the squamous and basal cell layers of the skin [1]. Among the keratinocyte cancers, basal cell carcinoma (BCC) is the most prevalent form, entailing roughly 5 million new cases annually in the US alone [2]. A major risk factor for BCCs is exposure to ultraviolet radiation, which leads to genetic mutations. In virtually all BCCs, these mutations cause dysregulation and increased activity of the hedgehog signaling pathway, which plays a pivotal role in BCC oncogenesis [3]. Hedgehog inhibitors (HHis) target the hedgehog pathway to decrease the expression of various proteins such as GLI family zinc finger 1 (GLI1), cyclins and MYC, which leads to reduced tumor cell survival, increased immune infiltration, and tumor remission [4,5,6,7,8,9] (see Figure 1). Of these three proteins, GLI1 is the most potent effector and mRNA levels of *GLI1* are often used to estimate biological response following HHi treatment [10].

Two smoothened (SMO) inhibitors, vismodegib and sonidegib, have been approved by the Food and Drug Administration (FDA) and European Medicines Agency (EMA) for systemic treatment of advanced and metastatic BCCs [11]. The efficacy of vismodegib and sonidegib after treatment of locally advanced BCCs for 18–21 months is 47.6% (30/63) and 60.6% (40/66), respectively [12]. However, during treatment, most patients experience adverse effects such as muscle spasms, alopecia, dysgeusia and weight loss, which are caused by systemic SMO inhibition, and lead to treatment termination and tumor regrowth [12,13,14,15,16]. To improve HHi treatment, topical delivery methods that reduce systemic HHi exposure have been explored. Clinical trials show that topical HHi treatments are associated with fewer adverse effects allowing for new treatment opportunities [17,18]. For example, topical HHis could potentially be used for life-long treatment in patients with multiple BCCs, for combination therapy with other treatments, for prophylactic treatment of sun-exposed patients, and for adjuvant treatment of normal BCCs before excision [19].

An overview of potential candidates for topical HHi treatment is shown in Table 1. The size of HHis ranges from small molecules (0.1–1 kDa) to antibodies (150 kDa), where SMO inhibitors represent the most used HHis. HHis inhibit the hedgehog pathway in two ways, either directly by reducing the activity of hedgehog proteins like vismodegib [20], or indirectly by inhibiting cross-talk with other pathways such as imiquimod [21].

This review focuses on 12 HHis that directly target the hedgehog pathway and have been tested in clinical trials on BCCs (Table 1 and Table 2). These HHis include established HHis such as the FDA-approved vismodegib and sonidegib [11], experimental HHis such as patidegib and taladegib, and atypical HHis with other mechanisms of action such as itraconazole, which was originally developed as an antifungal [27], and vitamin D₃, whose role in BCC oncogenesis and treatment is complex [28]. The 12 HHis are advantageous drug candidates for topical delivery due to their lipophilicity and molecular weight of roughly 0.5 kDa [29,30], and sonidegib, patidegib, itraconazole, vitamin D₃ and CUR-61414 have all been tested in clinical trials for topical treatment of BCCs. Of these HHis, patidegib has reached the highest drug development stage by completing a phase III clinical trial in December 2020, but so far, no topical HHi has been approved for the treatment of BCCs.

To achieve successful topical delivery, three primary barriers must be overcome. First, sufficient intra-tumoral HHi concentrations need to be achieved, second, HHi treatment must lead to a biological response, and third, the biological response must persist long enough to produce a clinical response. The aim of this review was to evaluate the current status of topical HHi delivery for BCCs and discuss the barriers for translating systemic HHi treatment into topical treatment.

## 2. Results

### 2.1. Preclinical Studies

From our search, we identified 16 preclinical studies reporting on the effects of topical HHi application using either ex vivo models (*n* = 5), in vivo models (*n* = 5), or both in combination (*n* = 6). An overview of the studies is presented in Table 3. Vismodegib was investigated in the largest number of studies (*n* = 8), itraconazole in five studies, sonidegib and CUR-61414 in two studies and vitamin D_3_ in one study. LEQ506, BMS-833923, taladegib and TAK-441 were only included once in a study comparing multiple HHis [33]. Topical treatment in combination with skin pre-treatments such as microneedles (*n* = 3) and ablative fractional laser (AFL, *n* = 2) was also investigated.

In four of the preclinical studies, pre-treatment of the skin was included before topical application of HHi [38,39,40,45]. Olesen et al. tested AFL as a pre-treatment before application of vismodegib formulation in both ex vivo and in vivo pig skin. AFL treatment creates microscopic channels in the skin and was found to enhance vismodegib concentration after 24 h in ex vivo skin when compared to no pre-treatment [39]. In in vivo skin, AFL boosted vismodegib concentrations as early as 4 h after treatment with the highest increase observed after 5 days [40]. Similarly, microneedles also create channels in the skin before topical application. One study demonstrated that increased microneedle length and microneedle application time enhanced vismodegib penetration of the skin [38], and another study showed increased drug uptake following treatment with itraconazole containing dissolving microneedles [45]. In the latter study, itraconazole uptake was measured at multiple timepoints with the highest itraconazole concentration detected after 2 h in epidermis and 3 h in dermis. Furthermore, the study showed that itraconazole remained in the skin at least 72 h after treatment, especially in dermis [45]. Overall, pre-treatment with AFL and microneedles enhanced skin uptake of HHis.

#### 2.1.1. Drug Concentration in Skin

Eleven of the preclinical studies focused on drug uptake in the skin following topical treatment. Franz cell and Saarbrücken penetration model setups, which use ex vivo skin to simulate an in vivo skin barrier, were most common (*n* = 10). However, direct comparison between publications was challenging due to inconsistent reporting of experimental results. In three directly comparable studies, skin concentrations were measured in ex vivo skin samples after treatment with vismodegib in specialized formulations including microemulsion, nanoformulation, and polymeric micelle nanocarriers. The highest skin concentration of vismodegib was achieved by Olesen et al. in pig skin (66 µg/mL) [39], while the other studies measured six to eight times lower concentrations in human skin (6.4–8.4 µg/mL) [34,35,37]. However, this may be explained by Olesen et al. using a substantially higher vismodegib dosing (>500 µg/cm^2^ versus 86 and 12 µg/cm^2^). According to Graham et al., the plasma concentration of vismodegib is between 2–24 µg/mL during oral treatment of humans [47], which is similar to the range of concentrations achieved in the studies. In the remaining studies, vismodegib or itraconazole skin uptake was reported as a percentage of total drug permeated, as a skin retention percentage, or as flux through the skin [36,41,43,44]. All studies concluded that novel HHi formulations improve skin uptake or permeation.

#### 2.1.2. Biological Response to Topical HHi Application

Biological response to HHi treatment is often assessed by investigating the expression of *GLI1* to estimate the activity of the hedgehog signaling pathway [10]. Four of the preclinical studies measured murine *Gli1* mRNA levels, but only one study compared mRNA reduction with skin drug concentrations. In this study, mice were depilated to activate the hedgehog pathway and increase *Gli1* transcription [33]. These mice were then used as a model to evaluate different HHis in terms of drug concentration in skin and *Gli1* inhibition. The authors found that even though some HHis had comparable IC₅₀ values in cell inhibition studies, HHi skin uptake and *Gli1* reduction varied widely in the in vivo setting [33]. In similar murine skin-depilation experiments, Skvara et al. showed that *Gli1* mRNA levels were reduced by 95% after 8 days of single topical sonidegib applications [18], and Tang et al. reported a 62% reduction in *Gli1* mRNA levels after 3 days of single topical CUR-61414 applications and by 85% after 3 days of two applications [32]. Overall, this indicates that the type of HHi as well as the frequency and duration of applications correlate with biological response to topical HHi treatment. 

The most complex models in the preclinical studies used experimentally induced murine BCCs to investigate biological and tumor responses to topical HHi treatment. Topical application of CUR-61414 and vitamin D_3_ was tested in the same murine BCC model. Twenty-one days of topical CUR-61414 treatment resulted in reduced *Gli1* expression and significant tumor remission [32], and four days of vitamin D_3_ treatment led to reductions in *Gli1* expression and tumor proliferation as measured by Ki67 protein levels [46]. Topical application of sonidegib and itraconazole also demonstrated effects in murine BCC models. Topical sonidegib blocked the formation of basaloids in ex vivo murine tissue [18], and topical itraconazole with microneedle pre-treatment prevented human BCCs from forming in nude mice [42].

### 2.2. Clinical Trials

We identified seven clinical trials consisting of the following: two patidegib phase II trials and one itraconazole phase I trial from 2016; one vitamin D_3_ phase II trial from 2011; two sonidegib phase II trials from 2009; one CUR-61414 phase I trial from 2005. An overview of the clinical trials is presented in Table 4. None of the clinical trials reported on the skin concentration of HHi, whereas the biological response (*GLI1* mRNA) was explored in four trials, and the clinical tumor response was investigated in all seven trials.

#### Biological and Clinical Response

As in the preclinical studies, biological response is estimated by *GLI1* mRNA expression, whereas clinical response is based on both objective and subjective measures e.g., changes in tumor volume, versus visually determined tumor clearance. In trials investigating CUR-61414 and itraconazole, no significant change in *GLI1* mRNA levels was reported, which corresponded with an observed lack of clinical response to treatment [31,32]. Topical treatment with vitamin D_3_ also had no clinical effect, but *GLI1* mRNA levels were not investigated ([48] and National Library of Medicine (NLM), NCT01358045). In trials on topical treatment with 2% or 4% patidegib, one to two daily applications over 12–26 weeks led to clearance of palpable tumor tissue with only visible residual macular erythema in 26/86 (30.2%) tumors, whereas placebo led to an equal response in 9/37 (24.3%) tumors (NLM, NCT02762084 and NCT02828111). Similarly, two daily topical applications of sonidegib over 4–9 weeks resulted in a partial response of at least a single tumor in 30/34 (88.2%) of patients, while placebo led to partial response in 6/16 (37.5%) of patients ([18] and NLM, NCT01033019 and NCT00961896). However, in one of the sonidegib clinical trials, subsequent histological examinations revealed that tumor nests were still present in all partial (n = 5) and all complete responders (n = 3) [18]. Biological response to treatment was also investigated demonstrating that both sonidegib and patidegib treatment reduced *GLI1* expression. It is worth noting that in both the sonidegib and patidegib trials, only a few patients were included. This led to a considerable impact of outliers, in part because of slow BCC regression [18] and the spontaneous response of placebo-treated BCCs (NLM, NCT02762084 and NCT02828111). Pre-treatment of the skin in combination with HHis has not been tested in a clinical setting.

## 3. Methods

In June 2022, a literature search was conducted to identify publications on the topical delivery of HHis in both in- and ex vivo preclinical studies as well as clinical trials. The search included PubMed and ClinicalTrials.gov databases with no time limit on publication date. The full search queries are listed in Table 5. For the PubMed search, we included the HHis from Table 2, and search terms covering basal cell carcinoma and topical application. For the ClinicalTrials.gov search, we removed topical application from the terms, because relevant clinical trials were excluded. The searches returned a total of 287 PubMed entries and 57 clinicaltrials.gov entries, which were screened to identify 16 preclinical studies and 12 clinical trials fit for inclusion. However, five of the clinical trials have not published their findings; thus, we could only include seven clinical trials.

## 4. Discussion

Topical treatment of BCCs with HHis holds great potential. HHis are potent molecules that can be formulated to cross the skin barrier, and topical HHi treatments have been shown to significantly reduce activity of the hedgehog signaling pathway in both murine skin and BCC models. However, when these topical HHi treatments are translated into the clinic, the observed outcome is less efficacious, which is reflected by the fact that no topical HHis are currently approved for treatment of BCCs. The main barriers that prevent effective topical HHi treatment of human BCCs appear to be the insufficient penetration of tumor tissue, lack of biological response, and poor biodistribution or too short intra-tumoral HHi presence during topical BCC treatment.

Insufficient tissue penetration results in drug concentrations too low to affect the target tissue. Preclinical studies showed that advanced formulations and pre-treatments could significantly increase topical uptake in both porcine and human skin. However, intra-tumoral HHi concentrations following either topical or systemic HHi treatment have never been measured, and it is unclear whether topical treatments penetrate tumor tissue as efficiently as they penetrate skin tissue. Notably, larger clinical studies showed that topical BCC treatment with diclofenac, imiquimod, or a combination of 5-fluorouracil and cisplatin was more effective against superficial BCCs than nodular BCCs [48,49,50,51]. This could be a result of insufficient drug penetration in the nodular subtype due to morphological differences that increase tumor depth [52,53], or a result of genetic variation between the subtypes [54,55]. Similarly, topical HHi treatments likely face the same challenges, and future studies on HHis may benefit by addressing these challenges. For example, knowledge of intra-tumoral HHi concentrations would allow future studies to verify HHi penetration of the tumor tissue and improve our knowledge of HHi cutaneous pharmacokinetics. However, currently only plasma and excrement concentrations have been measured clinically following oral treatment with the FDA-approved SMO inhibitors sonidegib and vismodegib [47,56,57]. 

Sufficient drug uptake is linked to biological response, but in some cases, a preclinical biological response is not reflected in clinical studies. The preclinical studies showed that both depilation models and BCC tissue respond to HHi treatment, and that the frequency and duration of applications affected this response. However, in most cases, these preclinical results did not translate well into clinical trials. For example, topical CUR-61414, vitamin D₃ and itraconazole reduced *Gli1* levels and decreased tumor size in murine preclinical studies, but when the drugs went into clinical trials, none of the patients responded to treatment ([31,32,48] and NLM, NCT02735356 and NCT01358045). Because of the complex nature of cancers, this poor translatability could be due to differences in the immune system [58,59], skin structures [60,61] and the vascularization and extracellular matrix of skin and tumor tissues [62,63]. For example, studies have shown that vismodegib binds with high affinity to α-1-acid glycoprotein—a protein present in blood and interstitial fluid—which results in early steady-state levels of vismodegib during oral treatment [47,64]. In topical treatments, α-1-acid glycoprotein could potentially affect the biodistribution and cutaneous pharmacokinetics of vismodegib by decreasing levels of unbound drug and increasing vismodegib wash-out from skin or tumor tissue. Because α-1-acid glycoprotein is present in both murine and human tissues, and studies have shown that murine α-1-acid glycoprotein levels change with age and inflammation status [65], α-1-acid glycoprotein could affect translatability of vismodegib studies. Similarly, other HHis might be affected by factors that change drug wash-out or biological response. For example, in ex vivo human percutaneous absorption studies, CUR-61414 concentrations far exceeded the IC_50_ levels, but when it was tested in clinical trials, no downregulation of *GLI1* mRNA was observed [32]. While drug potencies from in vitro experiments rarely translate directly into in vivo experiments, intra-tumoral CUR-61414 was not measured, which means that whether they achieved sufficient intra-tumoral concentration of CUR-61414 is unknown.

Apart from insufficient intra-tumoral HHi concentrations, a poor translation of biological response into clinical trials could also be associated with differences in tumor immune infiltration. Studies have shown that hedgehog inhibition leads to increased immune infiltration in BCCs [7,8,9], and that HHis can reduce the activity of regulatory T-cells [66], which are abundantly present in and around BCCs [67]. Currently, it is not known whether this immune regulation requires systemic hedgehog inhibition, e.g., in tumor-draining lymph nodes. This could explain why topical HHi treatments perform better in mice, in which a relatively large skin area is treated, potentially leading to some degree of systemic HHi distribution. Future studies should address HHi-induced immune regulation for topical treatments.

After a biological response to HHi treatment is established, it must persist for long enough to induce a clinical response. Trials on oral vismodegib show that the median time to response for advanced BCCs is around 15–20 weeks [13]. Comparably, the median length of the included topical HHi clinical trials was 7 weeks (IQR 4 to 12 weeks), while the longest trial was 26 weeks. Therefore, the included trials might not last long enough to achieve a clinical response. However, some patients showed a clinical response to topical sonidegib already after 6 weeks (NLM, NCT01033019), which suggests that factors other than time-on-target alter the BCC response. These factors could include drug resistance of some tumor cells, intermittently insufficient drug penetration, or a combination of the two. If intra-tumoral HHi concentration drops too low between topical applications, HHi biodistribution might suffer, which could explain why tumor nests remained in patients where treatment appeared successful [18]. On the other hand, even though HHi concentration is sustained at steady state during oral treatment of advanced BCCs [47], stable disease or tumor regrowth after treatment termination is commonly observed [14]. Future clinical studies on topical HHis will have to investigate whether HHi resistance is common in non-advanced BCCs, which in turn will help decide whether topical HHi treatment is best suited for monotherapy, adjuvant therapy to surgery, or combination therapy with other established topical BCC therapies.

The preclinical studies showed that skin pre-treatments improve the cutaneous uptake of HHis. Thus, future topical HHis treatments might benefit from the inclusion of pre-treatments. However, as HHis need extended time-on-target, potential pre-treatments must be repeatable without significant adverse effects to maintain sufficient HHi concentrations during treatment. Since repeated pre-treatments were not investigated in preclinical studies and the clinical trials did not include pre-treatments at all, there is a knowledge gap of whether these topical delivery methods can be applied at sufficient frequency to improve HHi delivery. Inclusion of pre-treatments may also reduce patients’ ease-of-use and raise treatment costs if the pre-treatment needs to be applied by a physician. Overall, the ideal topical HHi treatment would be able to sustain long-term concentrations of HHi in BCCs without significant increases to cost, treatment time and the number of medical checkups.

## 5. Conclusions

Preclinical studies focused on HHi uptake in pig and human skin and biological response in murine models. The studies demonstrated that topical delivery of HHis can be improved and that topical HHi treatment leads to biological response of the hedgehog pathway in murine skin and tumor models. However, when the topical treatments were translated into a clinical setting, they had little or no effect on BCCs. We find that the main barriers that prevent clinical response to topical HHi treatment include insufficient drug penetration and a lack of biological response due to the poor translatability of preclinical studies. Furthermore, partially successful clinical trials are limited by incomplete understanding of cutaneous pharmacokinetics, HHi biodistribution and biological response over time. Overall, novel topical delivery techniques could have the potential to improve HHi delivery, but additional knowledge of cutaneous pharmacokinetics and biological response of BCCs is necessary to guide further development.

## Figures and Tables

**Table 1:** Overview of different hedgehog inhibitors (HHis) and their molecular target. We selected HHis by first determining whether the HHis have been tested on BCCs in a clinical trial followed by investigation of whether the HHi has a direct effect on the HH pathway. Empty cells indicate that the drug was excluded in previous steps.

Target	Drug	Drug Aliases	Tested on BCC in Clinical Trial?	Direct Effect on HH Pathway?	Included in the Review?	Reference
SMO	Vismodegib	GDC-0449	Yes	Yes	Yes	[5,22,23]
SMO	Sonidegib	Erismodegib, LDE225	Yes	Yes	Yes	[5,22,23]
SMO	Itraconazole		Yes	Yes	Yes	[22,23]
SMO	Patidegib	Saridegib, IPI-926	Yes	Yes	Yes	[5,22,23]
SMO	Vitamin D₃	Cholecalciferol, Calcitriol	Yes	Yes	Yes	[22]
SMO	CUR-61414		Yes	Yes	Yes	[5,23]
SMO	BMS-833923	XL-139	Yes	Yes	Yes	[5,23]
SMO	LEQ506		Yes	Yes	Yes	[22,23]
SMO	TAK-441		Yes	Yes	Yes	[5,22,23]
SMO	Taladegib	LY2940680	Yes	Yes	Yes	[5,23]
SMO	ZSP1602		Yes	Yes	Yes	[24]
GLI	Arsenic Trioxide		Yes	Yes	Yes	[5,23]
GLI	Imiquimod		Yes	No		[5]
SMO	Tazarotene		Yes	No		[22]
SMO	Acitretin		Yes	No		[22]
GLI	4SC-202	Domatinostat	No			[23]
GLI	GANT58		No			[5]
GLI	GANT61		No			[5,23]
GLI	Glabrescione B		No			[5,23]
GLI	NanoHHI (HPI-1)		No			[25]
GLI	Nanoquinacrine		No			[5]
GLI	Pirfenidone		No			[5,23]
GLI	Pyrvinium		No			[5]
GLI	HPI 1–4		No			[5]
HH ligand	3H8	MEDI-5304	No			[23]
HH ligand	5E1 antibody		No			[5]
HH ligand	Robotnikinin		No			[5]
HHAT	RU-SKI 41		No			[23]
HHAT	RU-SKI 43		No			[23]
SMO	ALLO-1		No			[23]
SMO	AZD8542		No			[23]
SMO	Cyclopamine		No			[5,23]
SMO	DCBCO1303		No			[26]
SMO	DHCEO		No			[23]
SMO	DY131		No			[23]
SMO	Glasdegib	PF-04449913	No			[5,23]
SMO	Jervine		No			[5]
SMO	MK-4101		No			[23]
SMO	MRT-83		No			[23]
SMO	PF403	CAT3	No			[23]
SMO	PF-5274857		No			[23]
SMO	Posaconazole	Noxafil, SCH56592	No			[23]
SMO	SANT-1		No			[23]
SMO	SEN450		No			[23]
SMO	Tretinoin		No			[22]

Abbreviations: BCC, basal cell carcinoma; GLI, GLI family zinc finger 1; HH, hedgehog; HHAT, hedgehog acyltransferase; SMO, smoothened.

**Table 2:** HHi drug structures and molecular properties. Information on ZSP1602 and arsenic trioxide was not found. An increased clogP value corresponds to increased lipophilicity.

HHi	MW [g/mol]	cLogP	Drug Development Stage	Molecular Structure	Reference
Vismodegib	421.3	3.8	FDA approval, oral treatment Indication: laBCC, mBCC No topical clinical trials	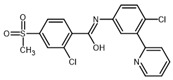	[11]
Sonidegib	485.5	5.8	FDA approval, oral treatment Indication: laBCC Phase II clinical trial, topical treatment of BCC	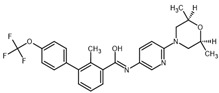	[11] and NLM, NCT00961896
Patidegib	504.8	4.6	Phase III clinical trial, topical treatment of BCC	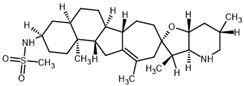	NLM, NCT03703310
Itraconazole	705.6	5.7	Phase II clinical trial, topical treatment of BCC	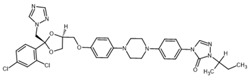	[31] and NLM, NCT02735356
Vitamin D₃	384.6	7.9	Phase II clinical trial, topical treatment of BCC	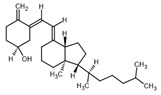	NLM, NCT01358045
CUR-61414	550.7	3.3	Phase I clinical trial, topical treatment of BCC	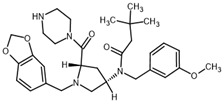	[32]
BMS-833923	473.6	5.7	Phase I clinical trial, oral treatment of BCC	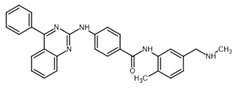	NLM, NCT00670189
LEQ506	432.6	2.9	Phase I clinical trial, oral treatment of BCC	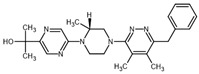	NLM, NCT01106508
TAK-441	576.6	2.6	Phase I clinical trial, oral treatment of BCC	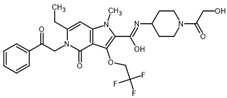	NLM, NCT01204073
Taladegib	512.5	4.3	Phase I clinical trial, oral treatment of BCC	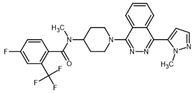	NLM, NCT01226485
ZSP1602	--	--	Phase I clinical trial, oral treatment of BCC	--	NLM, NCT03734913
Arsenic trioxide	197.8	--	Phase II clinical trial, IV injection treatment of mBCC	--	NLM, NCT01791894

Abbreviations: FDA, Food and Drug Administration (US); laBCC, locally advanced BCC; mBCC, metastatic BCC; MW, molecular weight; NLM, National Library of Medicine. MW and cLogP data have been retrieved from PubChem.

**Table 3:** Overview of included preclinical studies.

HHi	Formulation & Pre-Tx	Study Design	Delivery Method	Measurement Time	Effects	Reference
Vismodegib	Nanoformulation No pre-treatment	Ex vivo: Human skin	SPM	1 h, 4 h, 8 h	Human viable epidermis + dermis, 8 h: [8.4 µg/mL]	[34,35]
		In vitro: Human cell culture In vivo: Zebrafish larvae	Added to medium	4 h, 24 h, 48 h, 72 h	Tumor cell viability ↓ Larvae toxicity ↓	
	Binary ethosomes No pre-treatment	Ex vivo: Rat skin	Frz.C.	Running measure, 0 h to 24 h	Rat skin, 24 h: 40% of initial vismodegib permeated. Permeation flux: [3.22 ± 0.02 μg/cm^2^/h]	[36]
		In vivo: Mouse tumor skin	Topical app. 3 tx/week	Maybe 16 w	Tumor viability ↓	
	Polymeric micelle nanocarriers No pre-treatment	Ex vivo: Porcine skin, human skin	Frz.C.	6 h, 12 h, 24 h	Human skin, 120–200 µm depth, 12 h: [6.4 ± 3.3 µg/mL]	[37]
	Propylene glycol Microneedles (500, 1200, 1500 µm)	Ex vivo: Porcine skin	Frz.C.	Running measure, 0 h to 24 h	Increased needle length and needle app. time leads to enhanced penetration of vismodegib	[38]
	Oil/water microemulsion Ablative fractional laser	Ex vivo: Porcine skin	Frz.C.	0.5 h, 4 h, 24 h	Pig skin +AFL, 0–300 µm, 4 h: [85 µg/mL] Pig skin +AFL, 600–900 µm, 4 h: [35 µg/mL] Pig skin -−AFL, 0–300 µm, 4 h: [66 µg/mL] Pig skin -−AFL, 600–900 µm, 4 h: [37 µg/mL]	[39]
	Oil/water microemulsion Ablative fractional laser	In vivo: Porcine skin	Topical app. 1 tx	4 h, 2 d, 5 d, 9 d	Pig skin +AFL, 0–300 µm, 4 h: [131 µg/mL] Pig skin +AFL, 600–900 µm, 4 h: [30 µg/mL] Pig skin -−AFL, 0–300 µm, 4 h: [16 µg/mL] Pig skin -−AFL, 600–900 µm, 4 h: [6 µg/mL]	[40]
Sonidegib	Propylene glycol + ethanol No pre-treatment	Ex vivo: Murine basaloids	Added to medium	8 d	4 x fewer basaloid lesions	[18]
		In vivo: Porcine skin	Topical app. 1 tx	1 h to 8 h	Pig skin sonidegib concentration between 1 h and 8 h: [1–1.5 µg/g tissue]	
		In vivo: Murine hair regrowth	1 tx/d	15 d	Hair growth inhibited for 15 days	
		In vivo: Depilated murine skin	1 tx/d	8 d	Skin *Gli1* mRNA level ≈ −95% Skin *Gli2* mRNA level ≈ −87%	
Itraconazole	Nonionic surfactant vesicles No pre-treatment	Ex vivo: Rat skin	Frz.C.	1 h, 2 h, 3 h, 4 h, 6 h	Rat skin, flux: [1.88 ± 0.24 mg/cm^2^/h]	[41]
		In vivo: Tinea Pedis rat model	Topical app. 1 tx/d	14 d	Tinea Pedis infection is cured by both formulation and control itraconazole cream	
	DMSO + PEG Polyglycolic acid microneedles	In vivo: Human BCC regenerated in mice	Topical app. 1 tx/d	14 d	BCC formation seen in control group not present in treated mice	[42]
	Lipid nanocapsules No pre-treatment	Ex vivo: Human skin	Frz.C.	6 h	Itraconazole skin retention at 6 h: 66.3 ± 2.5%	[43]
		In vivo: Cutaneous candidiasis, rat skin	Topical app. 2 tx/d	14 d	Both novel and control treatments cure candidiasis infection	
	Nanoemulsion No pre-treatment	Ex vivo: Mouse skin	Frz.C.	6 h	27.6 ± 4.4% of itraconazole permeated after 6 h. 72.9% was present in skin or permeated at this time point	[44]
	Nanocrystals Microneedles	Ex vivo: Porcine skin	Frz.C.	0.5 h, 1 h, 2 h, 3 h, 4 h, 5 h, 6 h, 8 h, 12 h, 24 h, 48 h, 72 h	Highest concentrations reached in dermis after 3 h [1.97 ± 0.32 mg/cm^3^]. Drug diffuses deeper than needle length.	[45]
		Ex vivo: Candidiasis infection, porcine skin	Skin sustained in a Frz.C.	12 h, 24 h, 48 h, 72 h	Microneedle treatment cure candidiasis infection after 48 h, control cream only shows limited effect	
Vitamin D₃	Acetone No pre-treatment	In vivo: Murine BCCs	Topical app. 1 tx/d	30 d	Lower proliferation of treated BCCs but no cell death.	[46]
		In vivo: Murine BCC *Gli1* mRNA		4 d	BCC *Gli1* mRNA level ≈ −66%	
CUR-61414	Topical formulation No pre-treatment	In vivo: Depilated murine skin *Gli1* mRNA	Topical app. 1–2 tx/d	3 d	2 tx/d, skin *Gli1* mRNA level ≈ −85% 1 tx/d, skin *Gli1* mRNA level ≈ −62%	[32]
		In vivo: Murine BCC *Gli1* mRNA	10 tx/w	21 d	BCC *Gli1* mRNA level ≈ −60–65%	
Multiple	Propylene glycol + DMSO or Propylene glycol + DMSO + ethanol No pre-treatment	In vivo: Depilated murine skin	Topical app. 1 tx	8 h	Highest topical inhibition by LY-2940680, *Gli1* mRNA: −85% Vismodegib, *Gli1* mRNA: −40% Sonidegib, *Gli1* mRNA: −60%	[33]

Abbreviations: app., application; BCC, basal cell carcinoma; DMSO, dimethyl sulfoxide; Frz.C., Franz cell; PEG, polyethylene glycol SPM, Saarbrücken penetration model; tx, treatment(s).

**Table 4:** Overview of included clinical trials.

HHi	Formulation & Pre-Tx	Study Design	Delivery Method	Measurement Time	Effect(s)	Reference
Sonidegib	Topical formulation No pre-treatment	Clinical trial: Phase II Superficial or nodular BCCs. n = 24 BCCs	Topical app. 2 tx/d	6 w	0.75%, complete regression: 3/16 0.75%, partial regression: 9/16 0.75%, no reaction: 4/16	NCT01033019
	Topical formulation No pre-treatment	Clinical trial: Phase II BCNS patients, n = 61 BCCs	Topical app. 2 tx/d	4 w, 6 w, 9 w	Tumor volume ± SD: 4 w, 0.75%: −53,4 ± 30.85% 6 w, 0.25%: −35.2 ± 37.99% 9 w, 0.75%: −61.3 ± 31.18%	NCT00961896, [18]
Itraconazole	Topical formulation No pre-treatment	Clinical trial: Early phase I BCNS patients and high frequency BCCs. n = 79 BCCs	Topical app. 2 tx/d	4 w, 12 w	No effect on BCC (*GLI1* mRNA levels and tumor size) Intra-tumoral drug concentration: 4 w: [133 μg/g]; 12 w: [96 μg/g]	NCT02735356, [31]
Patidegib	Topical formulation No pre-treatment	Clinical trial: Phase II BCNS patients. n = 85 BCCs. 5–6 patients per group with multiple treated tumors	Topical app. 2 tx/d	26 w	*GLI1* mRNA levels ± SD: Patidegib 2%: [−54 ± 27%]; 4%: [−21 ± 35%] Tumor size ± SD: Patidegib 2%: [−51 ± 42%]; 4%: [−27 ± 41%]	NCT02762084
	Topical formulation No pre-treatment	Clinical trial: Phase II Nodular BCCs. n = 38 BCCs. 6 patients per treated group, multiple tumors per patient	Topical app. 1–2 tx/d	12 w	*GLI1* mRNA levels ± SD: 1 tx/d, 2%: [−56 ± 99%]; 4%: [−3 ± 69%] 2 tx/d, 2%: [−43 ± 56%]; 4%: [−29 ± 46%] Tumor size (±SD): 1 tx/d, 2%: [+56 ± 48%]; 4%: [+9 ± 47%] 2 tx/d, 2%: [+17 ± 37%]; 4%: [+18 ± 61%]	NCT02828111
Vitamin D₃ & diclofenac	Topical formulation No pre-treatment	Clinical trial: Phase II Superficial or nodular BCCs. n = 64	Topical app. 2 tx/d	8 w	No effect on BCC (clinical response)	NCT01358045, [48]
CUR-61414	Topical formulation No pre-treatment	Clinical trial: Phase I Superficial or nodular BCCs. n = 42	Topical app. 2 tx/d	4 d	No effect on BCC (*GLI1* mRNA levels)	[32]

Abbreviations: app., application; BCC, basal cell carcinoma; BCNS, basal cell nevus syndrome (also Gorlin syndrome); SD, standard deviation; tx, treatment(s).

**Table 5:** Search strategy. All search queries used for our searches. Cutane* covers all words that start with “cutane” e.g., cutaneous, and cutaneously. The latest search was performed 10 June 2022.

PUBMED		
Search	Query	Hits
1	(BMS-833923 OR XL-139) OR (“CUR 61414”) OR (Itraconazole) OR (LEQ506) OR (Patidegib OR Saridegib OR IPI-926) OR (Sonidegib OR Erismodegib OR LDE225) OR TAK-441 OR (Vismodegib OR GDC-0449 OR HhAntag691) OR (“Vitamin D3” OR Cholecalciferol OR Calcitriol)	52,838
2	#1 AND (“basal cell carcinoma” OR BCC OR (“Skin/abnormalities” [Mesh] OR “Skin/adverse effects” [Mesh] OR “Skin/cytology” [Mesh] OR “Skin/drug effects” [Mesh] OR “Skin/organization and administration” [Mesh] OR “Skin/pharmacology” [Mesh] OR “Skin/surgery” [Mesh] OR “Skin/therapeutic use” [Mesh] OR “Skin/therapy” [Mesh]))	1115
3	#2 AND (topical OR “Administration, Topical” [Mesh] OR cutane* OR “transdermal”)	287
CLINICALTRIALS.GOV		
Search	Query	Hits
1	Condition or disease: BCC OR basal cell carcinoma Other terms: (BMS-833923 OR XL-139) OR (“CUR 61414”) OR (Itraconazole) OR (LEQ506) OR (Patidegib OR Saridegib OR IPI-926) OR (Sonidegib OR Erismodegib OR LDE225) OR TAK-441 OR (Vismodegib OR GDC-0449) OR (“Vitamin D3” OR Cholecalciferol OR Calcitriol)	57

## Data Availability

Not applicable.
